# Assessment of the Risk of Failure in Electric Power Supply Systems for Railway Traffic Control Devices

**DOI:** 10.3390/s25144501

**Published:** 2025-07-19

**Authors:** Tomasz Ciszewski, Jerzy Wojciechowski, Mieczysław Kornaszewski, Grzegorz Krawczyk, Beata Kuźmińska-Sołśnia, Artur Hermanowicz

**Affiliations:** 1 Department of Computer Science and Data Communication Technologies, Faculty of Transport, Electrical Engineering and Computer Science, Casimir Pulaski Radom University, Malczewskiego 29, 26-600 Radom, Poland; beata.kuzminska-sols@urad.edu.pl (B.K.-S.); artur.hermanowicz@urad.edu.pl (A.H.); 2 Department of Electrical and Power Engineering, Faculty of Transport, Electrical Engineering and Computer Science, Casimir Pulaski Radom University, Malczewskiego 29, 26-600 Radom, Poland; j.wojciechowski@urad.edu.pl (J.W.); g.krawczyk@urad.edu.pl (G.K.); 3 Department of Control Systems and Electronics, Faculty of Transport, Electrical Engineering and Computer Science, Casimir Pulaski Radom University, Malczewskiego 29, 26-600 Radom, Poland; m.kornaszewski@urad.edu.pl

**Keywords:** power supply of railway traffic control equipment, safety, reliability model, Markov processes, maintenance processes

## Abstract

This paper provides a reliability analysis of selected components in the electrical power supply systems used for railway traffic control equipment. It includes rectifiers, controllers, inverters, generators, batteries, sensors, and switching elements. The study used failure data from power supply system elements on selected railway lines. The analysis was performed using a mathematical model based on Markov processes. Based on the findings, recommendations were made to improve safety levels. The results presented in the paper could serve as a valuable source of information for operators of power supply systems in railway traffic control, helping them optimize maintenance processes and increase equipment reliability.

## 1. Introduction

Rail transport is one of the most efficient and environmentally friendly forms of public transport. It stands out for its energy efficiency, minimal carbon footprint, and low negative environmental impact [1,2,3,4]. Nevertheless, for rail to be considered a viable alternative to other modes of transport, a high operational quality level must complement its environmental advantages. Key aspects include the speed of travel; the comfort of travel; and, above all, the safety of passengers and the goods being transported [5,6,7].

Risk mitigation of critical events, including accidents and train collisions, is contingent upon implementing effective railway traffic management. Contemporary railway traffic control and management systems (RCMS) are crucial for ensuring smooth and safe rail traffic operations. However, their effectiveness depends on reliability, and this can be threatened by the difficult operating conditions in which these systems often operate [8,9,10,11]. The constant development of railway traffic control technologies and care for high-quality infrastructure are crucial for further improving the safety and efficiency of rail transport.

The capacity of railway networks is becoming increasingly critical. Researchers [10,11,12,13] suggest that a significant step toward enhancing the capacity of railway lines involves modernizing railway traffic control devices and reducing the intervals between trains operating on the same line [14,15]. The literature also indicates the potential advantages of deploying modern transmission technologies along railway corridors to improve traffic efficiency [16]. Additionally, there is a recognized need to refine the diagnostic and monitoring mechanisms of such systems [17,18,19]. It is essential to ensure adequate power quality, as well as the reliability of power supply systems [20,21,22,23,24].

The challenges regarding the power supply for railway infrastructure equipment, including traffic control devices, have been discussed in sources [22,24,25,26]. These references address the quality of the power supply and the assessment of electrical quality parameters within system power supply circuits, specifically for railway signaling and interlocking systems [20,23,27]. Furthermore, other studies have explored phenomena associated with the power supply process and their environmental impact [1,2,4,22,26]. The importance of an uninterrupted power supply for railway traffic control devices during power system failures, alongside the causes of such failures and their implications for the safety of railway transport and traffic control systems, has also been discussed [21,28,29].

The issue of railway traffic safety, based on the proper functioning of railway traffic control systems, has been analyzed in studies [11,15,17,30,31,32], while the assessment of transport system safety has been addressed in references [8,9,25,33,34], which highlight the technical and operational reliability of selected facilities (so-called bottlenecks) and the entire system.

**Figure 1:**
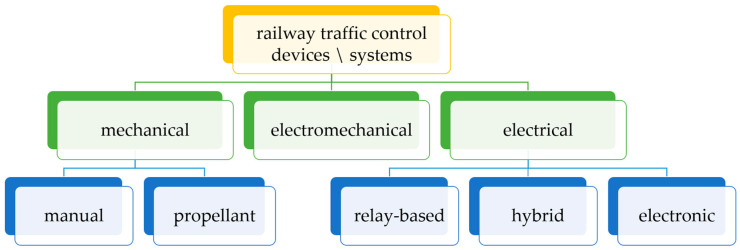
The classification of signaling devices according to how interlocking is implemented.

Railway traffic control is a set of actions, procedures, and technical measures designed to ensure the safety of rail vehicles moving along the rail network. The railway traffic control system is comprised of various subsystems and devices that are utilized for monitoring and controlling the positions of trains, as well as the operation of switches and signals, and the management of rail vehicle traffic [12,14,16,30,31,32,35,36]. The primary function of the signaling system is to ensure the safety of railway traffic; optimize the capacity of railway lines; minimize delays and improve punctuality; and efficiently manage railway assets such as tracks, switches, signaling, and level crossing protection systems [12,16,30,31,37]. RCMS devices are categorized in various ways, taking into account multiple criteria. The most characteristic and important is the division according to the following: the method of setting external devices, the implementation of interlocking, and the purpose of devices. The division of railway control devices according to the criterion of the realization of interlocking [30,31] is shown in Figure 1. In the process of railway traffic control, and therefore primarily train traffic management, the following are utilized (Figure 1):

- mechanical devices,
- electromechanical devices,
- electrical devices.

Electromechanical devices are those in which the interlocking is implemented both mechanically and electrically, while the control of external devices is executed electrically. The control elements of these devices, located in the control room, are connected to the switch machines in the area using cable networks. Some dependencies are established through dependency boxes, which contain slide-type devices, electromagnets, and relays. Electrical devices utilize independent electrical circuits for two main functions: securing a safe train route (by coordinating various trackside elements, such as signals, switches, and track circuits) and controlling the external equipment. As a result, the train routing time is reduced to a few seconds, enabling advanced automation of the routing process. Compared to mechanical and electromechanical devices, electrical devices have a significantly broader operational range, allowing for control of district implementation, such as covering the entire railway station.

The main components of the railway signaling system architecture are signaling systems, interlocking and dependences systems, remote control and operation systems, track clear detection testing systems (axle counters/wheel sensors, track circuits), level crossing protection systems, as well as power supply systems.

Sensors are essential components in modern railway traffic control systems. Their primary applications include the following [11,12,30,31]:

- Track Occupation and Train Detection: Inductive and magneto-inductive sensors are installed along the tracks to detect the passage of train axles, enabling precise tracking of a train’s location. This capability is critical for effective traffic management, establishing safe distances, and controlling signaling systems.
- Axle Counting and Train Integrity Checks: Modern track sensors enable the counting of each rail vehicle’s axle, as well as tracking travel speed and direction. This is essential for axle counting systems, which are used to control track and switch availability.
- Monitoring the State of Track Equipment: Position sensors are utilized to verify the positioning of railroad switches and derailers, along with the status of light signals. This monitoring allows for the automatic detection of failures or malfunctions within these components.
- Additionally, systems like the European Train Control System (ETCS) employ track balises to transmit real-time information about the current traffic situation to the train. This enables real-time control of driving parameters [16,32].

Ensuring safe and uninterrupted operation of railway traffic control systems is dependent on the integrity of power supply systems, which are a critical component of railway infrastructure [20,21,22,30,31,33]. The condition of power supply systems is continuously monitored through a network of sensors that assess power availability and the quality of grid parameters. The data collected from these sensors form the foundation for the operation of reliable power supply systems. These sensors track voltage, current, and the operational status of individual modules within the power supply system. This capability facilitates the quick detection of failures, ensuring the uninterrupted operation of railway traffic control devices. Advanced diagnostic systems leverage sensor data to analyze the performance of the power supply system, enabling effective energy management and the identification of deviations from the required power supply parameters [20,21].

The components enumerated above are susceptible to the effects of aging and the potential for failure. The causes of failures in the electrical power supply systems of the track equipment are varied [20,21,22,23,25,38]. These are primarily attributable to accidental external factors [12,20,21,23,27,28,38]. These failures can result in the interruption of the electricity distribution to the signal box or interlocking container, which houses the key electrical components that power the signaling and interlocking devices [23,26,27,28].

The properties of the materials of the construction of electrical and electronic components of power supply systems for railway traffic control devices are subject to changes during operation, caused by the environment and operating conditions. Changes in the properties of construction materials have the potential to induce premature wear or the failure of components. The most significant factors influencing changes in the properties of structural materials are mechanical loads, changing thermal conditions, changing atmospheric conditions, and the harmful effects of electric current. The wear of a part is a continuous and unintended process that occurs in the original state of the mass of a given part, the chemical composition, the material structure, and the state of stresses in the subsurface layer of the element. Wear and tear may be natural, occurring gradually during the device’s operations, or excessive, resulting from improper operation, faulty design, or the manufacturing technology of the part. Component failure is defined as a change in continuity or shape that is caused by accident or fatigue factors in the material. These factors may occur separately or simultaneously, which may be accompanied by material changes; changes in mechanical and electrical properties; and even violent phenomena (e.g., melting insulation), usually located in the vicinity of the damaged section [13,22,23,25,26,27,28,38].

Railway traffic control systems are equipped with redundant power supply mechanisms designed to mitigate common causes of power failure, thereby enhancing the reliability and continuity of operations in critical transportation infrastructure [39,40]. Grid failures and disturbances are among the most prevalent causes of system failures. These issues frequently manifest as voltage drops, surges, short circuits, overloads, waveform distortions, and electromagnetic interference, often stemming from traction and external systems like telecommunications. Each of these factors can directly or indirectly lead to the malfunction of devices and their power supply systems. Voltage converters, stabilizers, and other power supply components may suffer damage due to surges (from the mains or lightning), short circuits, overheating, and the effects of aging, particularly in batteries.

The quality of electronic components, along with thermal and environmental conditions (such as humidity, dust, and vibrations), significantly influences performance [41,42,43]. These disturbances can lead to memory damage, data loss in controllers and processors, and operational errors. Typically, these issues are addressed through the redundancy of control systems and decision-making algorithms, which help ensure that railway traffic control systems and devices maintain safe states.

Inverters and converters are susceptible to failures caused by electrical factors (power surges and lightning strikes), environmental factors (high temperature, humidity, dust), mechanical and operational factors (vibration, overload), as well as design and assembly defects and the wear and tear of components (e.g., capacitors, transistors) [43,44,45,46,47,48,49,50,51]. Additionally, programming errors and electromagnetic interference can negatively affect their operation [43,44].

Rectifiers can fail due to various technical and operational factors. The most prevalent causes include power surges and inadequate protection; improper operating conditions, such as overload and overheating, often resulting from insufficient cooling or contaminated heat sinks; operating errors like incorrect wiring, mechanical damage, ingress of moisture or contaminants, and the natural wear and tear of components; and issues related to connections quality and corrosion [42,43,52].

Current overloads, surges, and short circuits lead to damage to diodes and thyristors. Additionally, loose or faulty electrical connections increase resistance, leading to local overheating. The aging of components also contributes to a gradual loss of their properties. Assembly errors and improper RC snubber circuits further elevate the risk of failure [42,43,52].

Damage to surge protection devices (SPDs) can arise from a combination of technical and environmental factors, as well as human errors [53]. The most frequent causes include material aging, overheating, overloading, mechanical damage, and the poor technical condition of cables and connections. Environmental conditions such as lightning, high winds, icing, and moisture significantly contribute to these issues, potentially resulting in overvoltage, short circuits, and damage to insulation. Additionally, design and installation mistakes; improper selection of protective equipment—though this should not occur with certified devices intended for railway applications; insufficient maintenance; and improper use or overloading of circuits by users significantly increase the risk of failure [53,54].

Contact devices such as contactors, relays, and circuit breakers frequently fail due to several factors, including electrical overload [55,56,57], the wear and erosion of contacts [55,57], contamination, installation errors, vibration, adverse environmental conditions, and insufficient regular maintenance. Typically, failure is attributed to overheating [56], electric arcing [55], and loosening of contacts, which ultimately results in a loss of device efficiency [58,59].

The causes of failure obviously also include, among others, improper utilization and maintenance practices. Human errors play a critical role in this context, manifesting as design imperfections, unstable installations characterized by loose connections, irregular or inadequate maintenance, and delays in identifying early signs of wear [60,61].

Several important scientific studies [62,63,64,65,66] focus on the transport objects modeling, including railway traffic control devices and systems, by utilizing a formal description of the control process [7,38,67,68]. Publications [6,36,63] demonstrate that the relation between the reliability and safety of railway traffic control system structures, alongside the automation of the control process and the variety of tasks performed, is critically significant.

These models utilize Markov and semi-Markov process theory for this purpose. Each process, whether it involves a single element or a complex system, can be viewed as a multi-state Markov or semi-Markov process, among others [62,63,67,68]. Furthermore, the observed course of operation can be regarded as one of many possible realizations of this process. Formulating a random model to describe the actual exploitative process is a significant and complex challenge. To develop such a model, it is first necessary to identify the rules and dependencies governing this process, which comprises many factors. To ensure the effective management of the technical object in question, it is imperative to consider a multitude of factors. These include, but are not limited to, the intensity of the wear and tear of individual components and the system as a whole; its performance during different phases of wear and tear; the organization of its maintenance, including repairs, ongoing repairs, and maintenance; as well as numerous other aspects of the technical object’s operation process [63,64]. Various scholarly publications present applications for modeling the exploitation of specific transportation facilities in the context of disruptions caused by the inherent unreliability of these systems and improper operational practices [38,66,69,70].

In numerous countries, the ongoing renovation of machinery and equipment is a priority for manufacturers. They emphasize the importance of high quality; reliability; product functionality; a comprehensive range of replacement parts (including reconditioned options); and manageable operating costs, which encompass maintenance and repairs. In recent years, various concepts and models have emerged in the realm of technical facility maintenance, aiming to optimize the scheduling of maintenance tasks alongside a defined renovation strategy that aligns with specific economic and reliability criteria. The literature highlights traditional models for maintaining technical facilities, including the challenge of selecting the most suitable maintenance strategy [71,72]. In the context of railway traffic control systems, decision-making processes are crucial. The ability to articulate decision-making problems that consider real-world constraints for optimization, as well as strategies and operational policies, has been explored in various studies [33,35,62].

An important issue highlighted in studies [17,37] is the field of technical diagnostics, which involves developing processes for utilizing technical facilities. Technical diagnostics encompass a range of methods and measures aimed at evaluating the technical condition of a system, including its underlying causes, progression, and potential consequences. Typically, these systems are designed for specific purposes and are capable of generating or transforming information that can be utilized to assess their technical state. The necessity for diagnostics is underscored by models of object degradation, which consider factors such as the relation between wear and tear and the object’s age, the complexity of its construction, the advanced nature of its manufacturing technology, the intensity of its use, and the quality of its technical maintenance [64,65].

Issues related to the exploitation of power supply systems for railway traffic control devices also fall under the broadly understood research area. A significant issue that has been addressed in several publications is the impact of power failures on rail transport, including railway signaling devices [23,25,27,28]. In the contemporary context, the importance of reliability studies is becoming increasingly prominent. These studies, based on real data from diagnostic and monitoring systems [17,29,73], provide reliable information that is essential for the effective operation and maintenance of such systems. It appears evident that one must ascertain the fundamental quantitative and qualitative characteristics of power supply systems for railway traffic control devices, including but not limited to the mean time between failures, failure duration, and system availability.

## 2. Research Goal, Object, and Methodology

The objective of the research undertaken was to assess the risk of failures in electric power supply systems for railway traffic control devices. In addition, the research sought to analyze the most frequent failures in the power supply systems for railway traffic control devices. Furthermore, it was to identify probable causes of these failures and to propose possible preventive measures. The analysis included both internal and external factors that could affect the occurrence of failures. The data analysis was conducted based on the logged notifications for 12 railway lines. The railway lines comprised 33 routes and 54 stations and posts.

**Figure 2:**
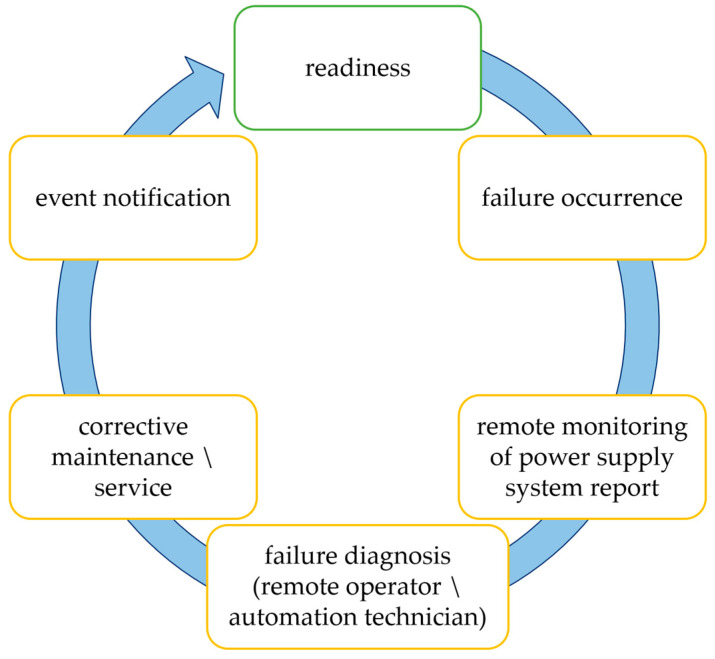
Schematic diagram of the troubleshooting process.

The troubleshooting process, as illustrated in Figure 2, is essential for effectively gathering detailed data needed to analyze the failure rate of power supply circuits for railway traffic control devices. In the event of a failure in the power supply system for the railway control devices, the controllers monitoring the correct operation of the devices shall report the failure to both the traffic controller’s operational position and the computer in the Maintenance and Diagnostics Center. The responsibility for handling submitted reports about the recorded failure lies with the designated rail automation equipment maintenance team. The automation technician responsible for maintaining the power systems is then tasked with analyzing the failure’s cause and, if he has the necessary resources, repairing it after reading the failure data from the diagnostic computer. In a scenario where on-site repair is not a viable option, the concerned employee is required to submit a report detailing the issue to the remote operator from the company that is responsible for the system’s warranty and/or maintenance. Subsequently, the remote operator logs the report into the designated system and, where practicable, provides instructions to a maintenance employee on how to rectify the failure. If on-site repair is still not possible, the report is forwarded to the service technician responsible for the given area. The service technician, equipped with the appropriate spare parts and detailed information about the failure, arrives at the site and removes the failure. After the repair is completed, a comprehensive protocol for the service intervention is formulated. This protocol encompasses a detailed description of the causes of the failure, the nature of the problem, and the repair activities that were undertaken.

An analysis was conducted on service intervention reports to ascertain the type and number of failures. The collected data (for 2022) are presented in Table 1.

**Figure 3:**
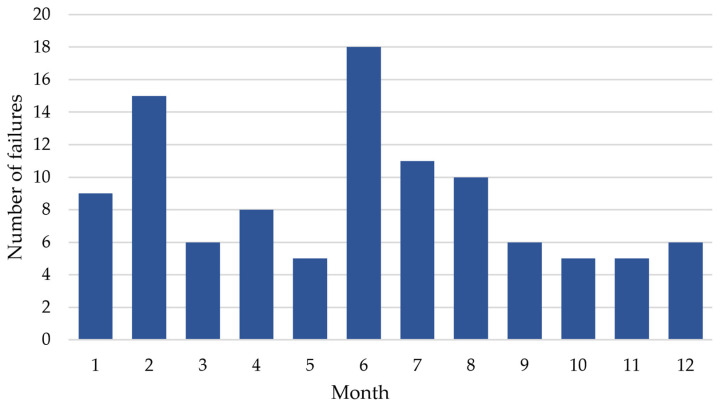
Monthly count of registered power supply failures for railway traffic control devices in 2022.

Figure 3 illustrates the number of registered failures related to the power supply for railway traffic control devices in 2022, disaggregated by month.

The highest number of failures was observed in June (18 failures) and February (15 failures). The elevated number of failures in February may be attributed to inclement winter conditions, while the increase in June may result from both high temperatures and the onset of the storm season. The summer months (June, July, and August) are characterized by a greater number of failures, suggesting that high temperatures and intense storms negatively impact the performance of power systems. A lower number of failures is noted in months such as May, October, November, and December due to more stable weather conditions and more efficient maintenance during these periods. Most failures were recorded for 48–60 V DC rectifiers, as well as those related to power failures or network interference. The fewest failures were observed in 230 V inverters. Rectifiers of various voltages, including 48–60 V, 24 V, and 120 V, experienced significant failures. Protection devices, power modules, and sensors also recorded a relatively high number of failures. Switching and terminal elements, damaged cables, and other categories of devices had fewer than 10 failures throughout the year.

Because of the considerable number of failures, it is essential that rectifiers operating at 48–60 V and 120 V DC are accorded the highest priority in maintenance activities and the identification of the root causes of these failures. It is clear that implementing a systematic inspection regime, accompanied by the replacement of components that show signs of wear and tear, and subsequently monitoring their operational functionality can help reduce the occurrence of failures. A detailed analysis of inverters is also advised to determine the root causes of any failures. It is conceivable that there is a necessity to enhance operating conditions and/or use more reliable components.

**Figure 4:**
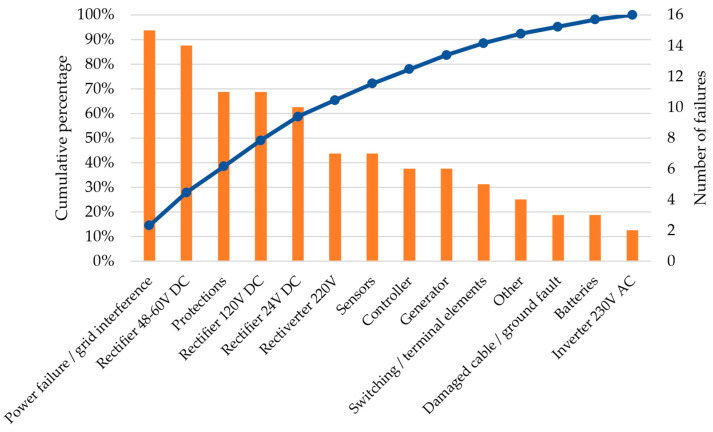
Pareto chart for the total number of failures in 2022.

A Pareto chart, as illustrated in Figure 4, is a tool used to identify and prioritize corrective actions. This can result in enhanced efficiency in managing power supply systems for railway control devices. The chart delineates the categories of devices and systems that are most prone to failure and require immediate attention. The emphasis on rectifiers, inverters, and power supplies is based on the understanding that such a focus can provide significant benefits, including a reduction in the number of failures.

Railway traffic control devices, which are crucial for ensuring safe operations, require a reliable power source to maintain continuous readiness. Currently, there are various technical solutions available in the market that meet the stringent requirements for powering both station and line railway traffic control systems. These solutions offer a comprehensive power supply for all necessary voltage levels. Depending on the specific characteristics of the facility and the available electricity sources, they are offered in several configurations:

- Dual power supply lines with a power generator,
- Single power supply line with a power generator,
- Dual power supply lines.

Each variant of the power supply system is designed to operate with an uninterruptible power supply (UPS). Furthermore, these solutions incorporate control and measurement functionalities, including the monitoring of voltage, current, phase sequence, and frequency. In standard configurations, ATS controllers manage these control functions. Nowadays, programmable PLC controllers are frequently utilized, complemented by a range of modular measurement systems. Together, these components create an advanced power supply system that includes supervision and diagnostics.

In the case of utilizing two independent power supply grids, the system is comprised of two low-voltage power lines. These lines derive their supply from distinct transformers, which function to reduce the medium voltage to 3 × 400/230 V. Importantly, these transformers receive input from separate high- or medium-voltage power lines, thereby enhancing the reliability and resilience of the overall power supply system.

Thanks to a modern and flexible design approach, along with the incorporation of new technologies, these systems can be used in railway traffic control that applies various technologies, e.g., computer, relay, and mechanical systems with light signaling. The system is usually designed to provide buffer charging for batteries and a backup power supply to selected technical rooms where railway traffic control devices are housed and where service personnel are active. Additionally, it powers other receivers such as wired and wireless communication devices, CCTV monitoring systems, and other auxiliary equipment that requires a continuous electricity supply.

For example, in Poland, devices are powered using connections from a separate non-traction power supply line (NTPSL). In such cases, a generator should serve as a backup power source, complemented by an uninterruptible power supply (UPS). If powering devices from the NTPSL is not feasible, it is acceptable to utilize power from a single power supply line for specific devices, such as automatic block signaling, level crossings, and siding and branch stations. Alternatively, two independent power networks may be employed to supply power to the station devices.

In a typical configuration, the power supply system delivers electricity to railway traffic control devices from the NTPSL, often referred to as the primary source. In the event of a power failure from this main source, the system automatically switches to a backup source, assuming dual power supply lines are part of the configuration. When the main network restores power, the system seamlessly switches back to the primary source. If power is not available from either of the supply grids, the system automatically switches to the power supply from the power generator. This automatic switching occurs only when the railway control devices are supported by a UPS power backup, ensuring that the change in the power supply network does not interrupt the devices’ power supply. Typical requirements for UPS backup duration range from 4 to 8 h.

In situations where devices lack an uninterruptible power supply (UPS) but are fitted with voltage converters—particularly, older-generation devices—a short-term power interruption may occur in circuits like traffic lights and other railway traffic control systems that do not utilize a converter. Furthermore, utilizing a power generator guarantees a consistent and reliable power supply.

**Figure 5:**
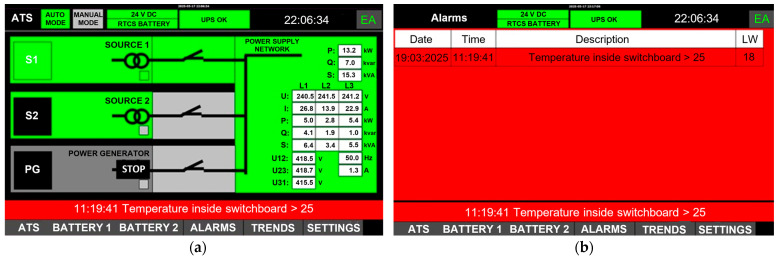
Network and generator panel: (**a**) operating status; (**b**) alarms.

The implementation of advanced automation and power supply metering typically enables comprehensive diagnostics and control over the operating parameters of devices, generators, and UPS systems (Figure 5a). Events and alarms associated with system components are logged in real time, and the solutions implemented facilitate remote monitoring of device operating parameters. In instances where operating parameters are exceeded or malfunctions arise, the system promptly notifies the user of the issue through visual and auditory alerts displayed on the control panel (Figure 5b).

**Figure 6:**
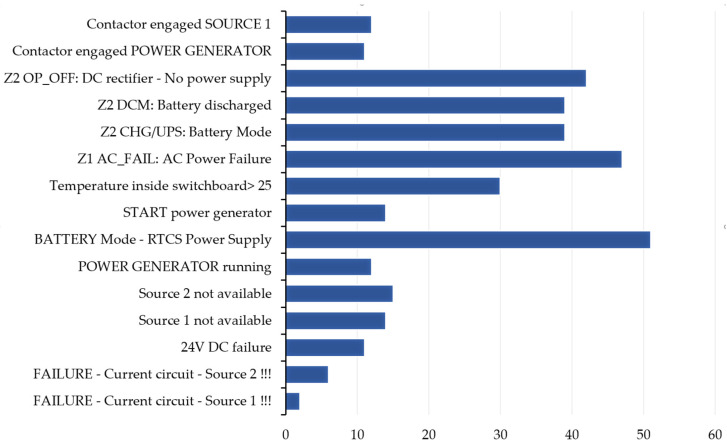
The alarm events logged in the smartATS system by type—object 1.

**Figure 7:**
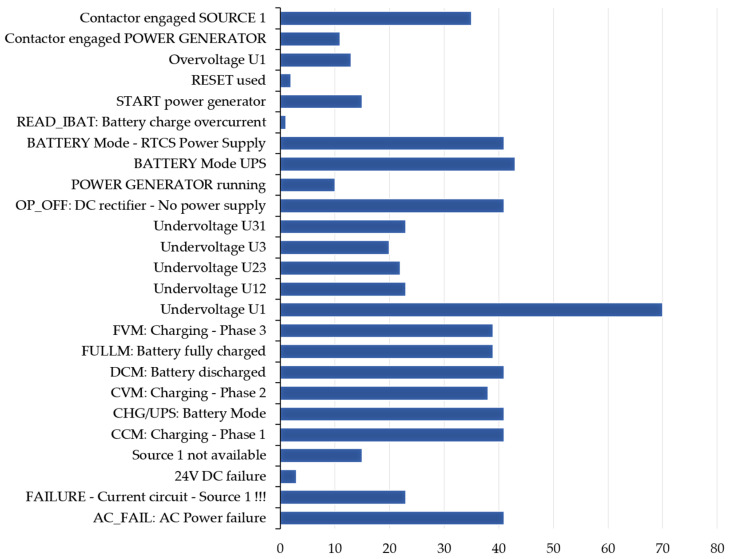
The alarm events logged in the smartATS system by type—object 2.

To explore the potential causes of events associated with power supply network failures, the authors examined internal data collected by the smartATS system from December 2024 to May 2025 for two facilities situated in different regions of Poland. The number of alarms logged for each facility is detailed in Table 2 and Table 3, while the cumulative data are illustrated in Figure 6 and Figure 7.

The data presented indicate that the power supply sources are not stable and fail-safe. An analysis of data collected from smartATS devices installed at two different locations within PKP, Polish Railway Lines, indicates the unreliability of the power supply networks. Notably, significant issues arise in December for both locations. The recorded events indicate challenges in meeting the requirements and ensuring the parameters of the power supply network. Frequently, the devices logged low-phase and interphase voltages.

## 3. Reliability Model of Electric Power Supply Systems for Railway Traffic Control Devices

The processes occurring in the operating power supply systems for railway traffic control devices, due to the randomness of the phenomena, can be classified as typical stochastic processes. A stochastic process is defined as a family of random variables that depend on a time parameter. Among the various classes of stochastic processes, Markov processes are of great importance from the point of view of the theory and modeling of safe systems. Markov processes are a tool for analyzing various technical objects [25,66]. Markov processes are also categorized as stationary stochastic processes. Stochastic process $X\left(t\right)$ is called a Markov process if for any finite system $t_{1}<t_{2}<\ldots <t_{n}$ parameter values and for any real numbers $x_{1},x_{2},\;\ldots \;,x_{n}$ [34]:

(1) $$P\left\{X\left(t_{n}\right)<\left.x_{n}\right|X\left(t_{n-1}\right)=x_{n-1},X\left(t_{n-2}\right)=x_{n-2},\ldots ,X\left(t_{1}\right)=x_{1}\right\}\;=P\left\{X\left(t_{n}\right)<\left.x_{n}\right|X\left(t_{n-1}\right)=x_{n-1}\right\}$$

where

*P*—probability of transition to the next state,

*X*(*t*)—stochastic process containing a set of states (a random variable representing a point in space—a value observed at time *t*),

*x_i_*—value of process *X*(*t*) at time *t_i_*.

Assuming the stationary, homogeneous, and ergodic nature of Markov processes, it is possible to model safety-related power supply systems. In order to facilitate the analysis of the power supply system, an indicator, in the form of system average availability *A*, was proposed. This indicator is a typical one for systems with repair. For such a system, the average availability can be defined as [34]:

(2) $$A=\underset{t\to \infty }{lim}\sum_{i=0}^{n}P_{i}(t)$$

where

*A*—average availability,

*P_i_*—probability that a system will operate satisfactorily at any given point in time *t* when used under its stated conditions (is in an operable and committable state).

**Figure 8:**
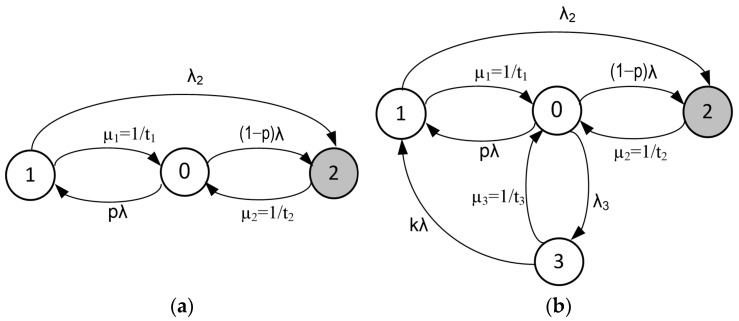
Markov model for power systems for railway traffic control devices. (**a**) Basic model for systems with repair; (**b**) Extended system model (with additional monitoring and diagnostic Subsystem). Where: p—probability of transition to a controlled failure state, *λ_i_*—intensity of failures, *µ_i_*—intensity of repairs (renewals), *t_i_*—mean repair time (MRT), *k*—probability of identifying a threat and transmitting a message for support.

The analysis was conducted using the proposed models depicted in Figure 8a,b. The first of these (a) is the classic model, which is most frequently used in the context of systems with repair. The second model (b) has been extended to include an additional monitoring and diagnostic system.

In both models, the following states can be distinguished:

- 0—an operable and committable state. There is no threat.
- 1—a controlled failure state. Despite the occurrence of the failure, the system retains the capacity to revert to an operational state without the necessity of external intervention.
- 2—a critical failure state. The system needs repair, and the intervention of a technician is required.

It was solely for model (b) that the additional status control state (3) was incorporated. The diagnostic system provides information about the system parameters.

An additional diagnostic subsystem, using an appropriate network of sensors, continuously monitors specific parameters of the power supply circuits within the railway traffic control devices, which could impact their reliability and, consequently, the safety of the entire system. These parameters include operating temperatures, humidity, fan speed, control system processor, and memory usage, among others. The information gathered from this subsystem is utilized to predict potential failures and provide early warnings for necessary maintenance.

For the models presented in Figure 8a,b, the equations can be expressed in operator form as follows:

(3) $$\left\{\begin{array}{l} s\cdot {\tilde{P}}_{0}-1=-p\cdot \lambda \cdot {\tilde{P}}_{0}+\mu_{1}\cdot {\tilde{P}}_{1}-\left(1-p\right)\cdot \lambda \cdot {\tilde{P}}_{0}+\mu_{2}\cdot {\tilde{P}}_{2}\\ s\cdot {\tilde{P}}_{1}=p\cdot \lambda \cdot {\tilde{P}}_{0}-\mu_{1}\cdot {\tilde{P}}_{1}-\lambda_{2}\cdot {\tilde{P}}_{1}\\ s\cdot {\tilde{P}}_{2}=\left(1-p\right)\cdot \lambda \cdot {\tilde{P}}_{0}-\mu_{2}\cdot {\tilde{P}}_{2}+\lambda_{2}\cdot {\tilde{P}}_{1} \end{array}\right.$$

(4) $$\left\{\begin{array}{l} s\cdot {\tilde{P}}_{0}-1=-p\cdot \lambda \cdot {\tilde{P}}_{0}+\mu_{1}\cdot {\tilde{P}}_{1}-\left(1-p\right)\cdot \lambda \cdot {\tilde{P}}_{0}+\mu_{2}\cdot {\tilde{P}}_{2}-\lambda_{3}\cdot {\tilde{P}}_{0}+\mu_{3}\cdot {\tilde{P}}_{3}\\ s\cdot {\tilde{P}}_{1}=p\cdot \lambda \cdot {\tilde{P}}_{0}-\mu_{1}\cdot {\tilde{P}}_{1}-\lambda_{2}\cdot {\tilde{P}}_{1}+\lambda \cdot {\tilde{P}}_{3}\\ s\cdot {\tilde{P}}_{2}=\left(1-p\right)\cdot \lambda \cdot {\tilde{P}}_{0}-\mu_{2}\cdot {\tilde{P}}_{2}+\lambda_{2}\cdot {\tilde{P}}_{1}\\ s\cdot {\tilde{P}}_{3}=\lambda_{3}\cdot {\tilde{P}}_{0}-\mu_{3}\cdot {\tilde{P}}_{3}-\lambda \cdot {\tilde{P}}_{3} \end{array}\right.$$

where

${\tilde{P}}_{i}$—denotes the probability of the i-th state of the system,

*λ*—intensity of failures,

*µ_i_*—intensity of repairs (renewals),

*s*—complex frequency variable in the s-domain.

The solution to the system of Equations (3) and (4) was found by utilizing the properties of the Laplace transform. The probabilities of critical failure states *P*_2_(*t*) were then calculated for the model in Figure 8a,b, as required for the calculation of each model’s availability (Equations (5) and (6)).

(5) $$A_{\left(a\right)}=1-\underset{t\to \infty }{lim}P_{2}\left(t\right)=\frac{\lambda \left(\mu_{1}-p\mu_{2}+\lambda_{2}\right)}{p\lambda \mu_{2}+\mu_{1}\left(\mu_{2}+\lambda -p\lambda \right)+\left(\mu_{2}+\lambda \right)\lambda_{2}}$$

(6) $$A_{\left(b\right)}=1-\underset{t\to \infty }{lim}P_{2}\left(t\right)=\frac{\left(\mu_{2}\left(\mu_{3}+k\lambda \right)\left(\mu_{1}+p\lambda +\lambda_{2}\right)+\mu_{2}\left(\mu_{1}+k\lambda +\lambda_{2}\right)\lambda_{3}\right)}{\left(\left(\mu_{3}+k\lambda \right)\left(p\mu_{2}\lambda +\mu_{1}\left(\mu_{2}+\lambda -p\lambda \right)+\left(\mu_{2}+\lambda \right)\lambda_{2}\right)+\left(\mu_{1}\mu_{2}+\mu_{2}\lambda_{2}+k\right)\right)}$$

The intensity of failure coefficients (*λ_i_*) was derived from the operational data that had been acquired, while the intensity of repair coefficients (*µ_i_*) was assumed to be the following values:

- *λ* = 0.00025 h^−1^ (intensity of failures estimated based on operational data for the studied area),
- *λ*_2_ = 0.000025 h^−1^ (intensity of transitions to a critical failure state constituting 10% of the λ value),
- *λ*_3_ = 60 h^−1^ (intensity of control by the diagnostic subsystem),
- *µ*_1_ = 6 h^−1^ (intensity of recovery—inverse of assumed recovery time = 10 min),
- *µ*_2_ = 0.33 h^−1^ (intensity of repair—inverse of assumed repair time = 3 h),
- *µ*_3_ = 60 h^−1^ (intensity of reporting—after 1 min, the diagnostic subsystem reports the system status—Figure 8b),
- *k* = 0.1 (probability of detecting a threat and sending a message to support).

The system’s availability A, according to the probability of a controlled failure state and the model used (see Figure 8a,b), is presented in Table 4.

The analysis results presented herein confirm the high availability of the power supply system for railway traffic control devices. The analysis, conducted utilizing the mathematical apparatus of Markov processes, indicates that the implementation proposed by the authors, an additional diagnostic system, enhances the readiness index. This is especially evident in instances where there is a high probability of critical failures.

## 4. Verification of the Assumptions for the Markov Model

A crucial component in evaluating the reliability of the control and management of power supply systems is assessing real systems based on operational data. One of the system verification and assessment methods is in-service testing, which provides an overview of the type of failures, intensity distribution, and the possibility of calculating characteristic indices. The operational data collected were analyzed, utilizing the electrical equipment data for the calculations, while excluding failures resulting from mechanical damage that were not directly related to the electrical equipment.

**Figure 9:**
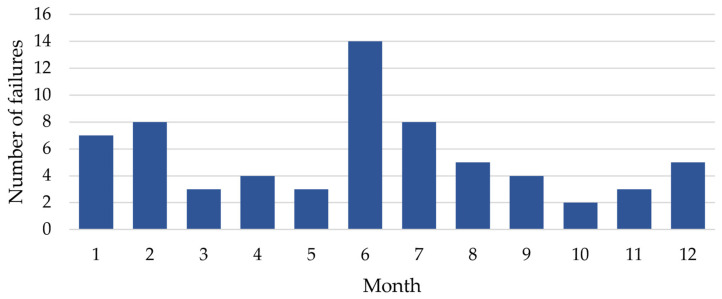
Number of selected failures used for the analysis of the Pearson χ2 test.

During the period under review, a total of 104 failures were documented. The χ2 (chi-squared) test was utilized to evaluate the nature of the durability distribution of the equipment related to the power supply for railway traffic control devices. The data obtained for the year 2022 have been segmented into six time slots. A total of 66 selected failures were deemed suitable for the subsequent analysis. As illustrated in Figure 9, a column chart is used to present failures qualified for analysis.

The data of the analysis are presented in Table 5.

The probabilities of each possible outcome *p_i_* were calculated using the following formulae:

(7) $$\begin{array}{l} p_{0}=0,\\ p_{i}=F\left({t^{\prime \prime }}_{i}\right)-F\left({t^{\prime \prime }}_{i-1}\right),\;\\ p_{r}=1-\sum_{i=1}^{r-1}F\left({t^{\prime \prime }}_{i}\right), \end{array}$$

where

*r*—assumed range (the number of time intervals).

For $\sum_{i=1}^{r}p_{i}=1$, the values of the cumulative distribution function in individual intervals were calculated based on the formula:

(8) $$F\left({t^{\prime \prime }}_{i}\right)=1-e^{\left(-\lambda \cdot {t_{i}}^{\prime \prime }\right)}$$

The distribution functions that have been calculated refer to the conclusion of the specified time interval. The intensity of the exponential distribution parameter was calculated using the following formula:

(9) $$\lambda =\frac{n}{\sum_{i=1}^{r}n_{i}t_{i\;avg}}$$

The quantile of the distribution is equivalent to the critical level of significance (α = 0.001) and the assumed range (*r* = 6):

(10) $$X_{r-1-v}^{2}=18.456$$

where

*r*—assumed range (the number of assumed intervals),

*ν*—number of unknown parameters of the hypothetical distribution F (for the exponential distribution, this parameter is equal to 1).

The obtained value $\chi_{emp}^{2}=17.296$ is smaller than $\chi_{r-1-v}^{2}=18.465;$ therefore, there is no basis to question the hypothesis that the failure times for the analyzed group of devices are related to the exponential distribution of failure times.

## 5. Discussion and Recommendations for Power Supply Systems

The analysis conducted has enabled the identification of measures aimed at enhancing the reliability of signaling power supply equipment, significantly contributing to rail safety. To improve the performance of pulse rectifiers, it is advisable to enhance the protection of the digital memory EPROM, optoisolators, JRC relays, and fuses with bases by using high-quality components. Overvoltage has been the primary cause of rectifier failure. The risk of rectifier failures resulting from overvoltages can be reduced by using protection in the form of installing a varistor between the phase and neutral conductors in the rectifier supply circuit, with the choice of varistor being appropriate for the system’s operating voltage. A second solution is to install a Zener diode with the appropriate protection voltage in the relay coil circuit to prevent overvoltages that can damage the coil. An additional option is to install an RC circuit in parallel to the relay contacts or coil. This will help to suppress the overvoltages created when the circuit is switched, which can extend the relay life and protect other components from overvoltages.

The installation of surge arresters at the rectifier input will ensure the protection of the entire system, including the relay and the fuse base. The SPD dissipates excess energy, thereby protecting the system from surges. It is also advisable to install a suppressor diode in parallel with the relay coil to prevent high reverse voltages that could damage the relay. Additionally, incorporating an EMI filter at the rectifier power input is recommended, as it helps shield the system from electromagnetic interference, which can lead to surges. The effects of such filters were also highlighted by the study [26] authors. It is crucial to ensure that the rectifier system is adequately grounded by established standards to safeguard components from the detrimental effects of excessive voltage. The process of grounding serves to dissipate excess energy, thereby safeguarding devices from the occurrence of surges. The installation of a transient voltage surge (TVS) diode in parallel with the relay and fuse base is recommended to mitigate surges caused by sudden voltage fluctuations. Finally, it is recommended that components of switching rectifiers be periodically inspected, with particular attention paid to the condition of the rectifiers and the monitoring of the operating devices’ conditions.

While a guaranteed power supply aims to protect railway traffic control systems from power loss impacts, it is not without risks and issues. Regular maintenance, testing, proper configuration, and redundancy are essential for these systems to ensure their effectiveness in maintaining operational continuity and railway traffic safety.

Power grid interference poses a serious threat to the railway traffic control devices’ power supply. To mitigate this risk and guarantee reliable operation, appropriate countermeasures must be implemented. The primary countermeasure recommended is the installation of EMI/RFI filters at the power supply system’s input, aimed at minimizing the interference introduced into the system by the power grid. Implementing appropriate selectivity of protection mechanisms, such as overcurrent and differential circuit breakers, allows for isolating only the components at risk of failure, thus preventing systemic shutdowns. Voltage stabilizers play a crucial role in maintaining the voltage within an optimal range, protecting the system against both insufficient and excessive voltage. The implementation of shielding for power and signal cables serves to minimize electromagnetic interference (EMI), which has the potential to affect voltage fluctuations in sensitive devices. Furthermore, surge arresters, when incorporated into railway traffic control power supply devices, offer an additional layer of protection against overvoltages resulting from lightning discharges. Advanced monitoring systems, combined with regular maintenance procedures, have been shown to enhance systems’ resilience to interference, thereby minimizing the risk of power supply disruptions to critical devices.

## 6. Conclusions

Railway traffic control devices are a key component in ensuring the safety of rail transport. Modern railway traffic control systems rely heavily on a comprehensive network of sensors. These sensors are essential not only for accurate diagnostics of the systems but also for ensuring the reliability and safety of train operations. Additionally, they play a crucial role in maintaining the reliability of power supply systems and, by extension, the entire infrastructure. The research results presented in this paper focus on a selected group of devices included in the supervision and control systems related to the electrical power supply for railway traffic control devices.

The research utilized statistical data regarding failures sustained by individual components. The analysis in the paper was conducted using a mathematical apparatus based on Markov processes. This apparatus enabled the determination of characteristic indicators associated with the safety and reliability of the power supply devices used in the RCMS systems. The developed models can serve as a foundation for creating systems designed to predict failures in power supply systems for railway traffic control devices. The incorporation of a more substantial dataset, covering extended observation periods, would facilitate enhanced modeling of real systems’ behavior. However, the model’s compliance with the data has already been confirmed through the implementation of an appropriate χ2 statistical test. A detailed investigation of the extended model (Figure 8b) with supplementary diagnostic states revealed that integrating additional monitoring and diagnostic subsystems into power supply systems for railway traffic control devices can reduce the failure rate of these systems.

The proposed concept introduces an extended power supply system that incorporates an additional monitoring and diagnostic subsystem. This subsystem analyzes specific operating conditions and evaluates the current performance of power supply systems for railway traffic control devices. It continuously monitors crucial parameters of the power supply circuits associated with railway traffic control devices that could impact their reliability and, consequently, the safety of the entire system. Key parameters, apart from power supply parameters, include, e.g., operating temperatures, humidity, fan speed, processor load of the control system, and memory usage. The data gathered from this subsystem are utilized to predict potential failures, enabling early alerts for necessary maintenance.

The authors did not explore the specific causes of the failure; instead, the reliability analysis primarily aimed to validate the central assumption of the publication. This assumption posits that the reliability of power supply subsystems for railway traffic control devices can be enhanced by integrating an additional monitoring and diagnostic subsystem.

The findings outlined in this paper can serve as a valuable source of information for operators of power supply systems for railway control devices, helping to optimize maintenance processes and improve device reliability. Power supply problems can result from various causes, such as failures of main power sources, power outages, or problems with the railway network infrastructure. Consequently, a comprehensive audit of power supply systems is imperative to identify and eliminate vulnerabilities. The paper also identifies recommendations that can increase the technical availability of power supply equipment.

## Figures and Tables

**Table 1:** The number of failures in power supply equipment for railway traffic control devices in 2022.

Devices	Number of Failures in Each Month												
	Σ	I	II	III	IV	V	VI	VII	VII	IX	X	XI	XII
Rectifier 48–60 V DC	14	2					8	2			1		1
Rectifier 24 V DC	10	2	3				3	1	1				
Rectifier 120 V DC	11	2	1	1	3	1	1		1	1			
Controller	6		1					2		1		2	
Electric protections	11	1	3				2		2	1	1		1
Inverter 230 V AC	2										1	1	
Rectiverter 220 V	7					2	1		1	2			1
Generator	6	1		1				1					3
Batteries	3				1			2					
Sensors	7		3	1			1		2				
Power failure (mains interference)	15		2	1	3	2	2	1	2			2	
Damaged cable (ground fault)	3			2						1			
Switching elements	5		1		1			1	1		1		
Other	4	1	1					1			1		
Total failures	104	9	15	6	8	5	18	11	10	6	5	5	6

**Table 2:** The alarm events logged in the smartATS system by month—object 1.

Alarm Label	Number of Alarms in Each Month						
	Σ	XII	I	II	III	IV	V
FAILURE—Current circuit—Source 1 !!!	2	2					
FAILURE—Current circuit—Source 2 !!!	6	5					1
24 V DC failure	11	1	7			3	
Source 1 not available	14	5	2	3	2		2
Source 2 not available	15	5	2	3	2	1	2
POWER GENERATOR running	12	3	2	3	2		2
BATTERY Mode—RTCS Power Supply	51	16	15	6	4	6	4
START power generator	14	7	2	3	2		
Temperature inside switchboard > 25	30	6	6	7	5		6
Z1 AC_FAIL: AC Power Failure	47	16	11	6	4	5	5
Z2 CHG/UPS: Battery Mode	39	16	6	6	4	3	4
Z2 DCM: Battery discharged	39	16	6	6	4	3	4
Z2 OP_OFF: DC rectifier—No power supply	42	16	6	6	4	5	5
Contactor engaged POWER GENERATOR	11	4	2	3	2		
Contactor engaged SOURCE 1	12	6	1			3	2
Total alarms	345	124	68	52	35	29	37

**Table 3:** The alarm events logged in the smartATS system by month—object 2.

Alarm Label	Number of Alarms in Each Month						
	Σ	XII	I	II	III	IV	V
AC_FAIL: AC Power failure	41	30	2	7		2	
FAILURE—Current circuit—Source 1 !!!	23	23					
24 V DC failure	3	3					
Source 1 not available	15	8	1	5		1	
CCM: Charging—Phase 1	41	30	2	6	1	2	
CHG/UPS: Battery Mode	41	30	2	7		2	
CVM: Charging—Phase 2	38	29	1	6		2	
DCM: Battery discharged	41	30	2	7		2	
FULLM: Battery fully charged	39	26	2	7	1	3	
FVM: Charging—Phase 3	39	26	2	7	1	3	
Undervoltage U1	70	15	13	18		2	22
Undervoltage U12	23	13	2	6		2	
Undervoltage U23	22	12	2	6		2	
Undervoltage U3	20	13	2	3		2	
Undervoltage U31	23	13	2	6		2	
OP_OFF: DC rectifier—No power supply	41	30	2	7		2	
POWER GENERATOR running	10	6	1	2		1	
BATTERY Mode UPS	43	32	2	7		2	
BATTERY Mode—RTCS Power Supply	41	30	2	7		2	
READ_IBAT: Battery charge overcurrent	1	1					
START power generator	15	8	1	5		1	
RESET used	2	2					
Overvoltage U1	13			13			
Contactor engaged POWER GENERATOR	11	7	1	2		1	
Contactor engaged SOURCE 1	35	28	1	5		1	
Total alarms	691	445	45	139	3	37	22

**Table 4:** The system’s availability A according to the probability of a controlled failure state and the model used.

Probability of Transition to a Controlled Failure State	Availability A Basic Model (a)	Availability A Extended Model with Additional Diagnostic Status (b)
0.99	0.999924	0.999962
0.7	0.997733	0.998865
0.5	0.996227	0.99811

**Table 5:** Data for calculations.

No	$\left(t_{i}^{\prime },t_{i}^{\prime \prime }\right)$		Average *t_i_*	*n_i_*	$F\left(t_{i}^{\prime \prime }\right)$	*p_i_*	*np_i_*	${\left(n_{i}{-np}_{i}\right)}^{2}$	χ2
1	0	1456	728	15	0.311253056	0.311253056	20.54270171	30.72154228	1.495496684
2	1456	2912	2184	7	0.525627647	0.214374591	14.14872302	51.10424083	3.611933088
3	2912	4368	3640	17	0.673277492	0.147649845	9.744889739	52.6366249	5.401459259
4	4368	5824	5096	13	0.774970871	0.101693379	6.711763025	39.54192426	5.891436291
5	5824	7280	6552	6	0.845011875	0.070041004	4.62270627	1.896938018	0.410352271
6	7280	8736	8008	8	0.893252403	0.154988125	10.22921623	4.969405009	0.48580506
Total						1			17.29648265

Where: *t_i_*′—beginning of the time interval, *t_i_″*—end of the time interval, *n_i_*—number of failures in the interval, *p_i_*—probabilities calculated according to Equation (7), *F(t_i_″)*—values of the cumulative distribution function calculated according to Equation (8), *n*—total number of failures, *χ*2—Chi-square test statistic.

## Data Availability

The original contributions presented in the study are included in the article; further inquiries can be directed to the corresponding author.

## Abbreviations

The following abbreviations are used in this manuscript:

RCMS	Railway Traffic Control and Management Systems
PLC	Programmable Logic Controller
CCTV	Closed Circuit Television
ATS	Automatic Transfer Switch
NTPSL	Non-Traction Power Supply Line
RTCS	Railway Traffic Control Systems
EPROM	Erasable Programmable Read-Only Memory
SPD	Surge Protection Device
EMI	Electromagnetic Interference
RFI	Radio Frequency Interference
TVS	Transient Voltage Surge
